# Perceived Impact of Social Media Use on Mental Health and Sleep-Related Outcomes Among Healthy Social Media Users: A Cross-Sectional Study

**DOI:** 10.3390/healthcare14121732

**Published:** 2026-06-16

**Authors:** Mohammed A. Aljunaid, Ruba Alghannami, Elaf Alshaikh, Abdulrahman Khalifa, Jood E Alzohari, Waad Alshamrani, Rahaf Alharbi

**Affiliations:** 1 Family and Community Medicine Department, University of Jeddah, Jeddah 23218, Saudi Arabia; 2 Faculty of Medicine, University of Jeddah, Jeddah 23218, Saudi Arabia

**Keywords:** social media, mental health, sleep quality, young adults, Saudi Arabia

## Abstract

**Background and objectives:** Social media use has become pervasive among the general population, with growing concern regarding its potential effects on mental health and sleep. While existing studies report associations between social media engagement and psychological outcomes, limited attention has been given to users’ self-perceived impact. To assess the self-perceived impact of social media use on mental health and sleep-related outcomes among healthy adolescents and adults aged 16–50 years old, and to identify associated demographic and behavioral factors. **Methods:** A cross-sectional survey was conducted among residents of Jeddah, Saudi Arabia, aged 16–50 years without a history of psychiatric or chronic sleep disorders, using a structured online questionnaire. Perceived mental health impact was assessed using a six-item study-specific questionnaire evaluating participants’ subjective perceptions regarding emotional and psychological responses to social media exposure. Higher perceived impact was defined as a composite score of 12–24 points on the study-specific scale. Data included sociodemographic characteristics, patterns of social media use, perceived mental health impact assessed through a 6-item Likert scale, and sleep-related outcomes. Associations were evaluated using chi-square tests and logistic regression analysis. **Results:** Most participants reported daily social media use exceeding 3 h, with 44.9% engaging in late-night use and 87.6% using devices within 30 min before sleep. Overall, 18.6% exhibited higher perceived mental health impact. Higher odds were observed among younger participants, students, and single individuals. Snapchat and YouTube use, and late-night engagement were independently associated with increased perceived impact. Approximately one-third reported insomnia after social media use, and 44.3% perceived improved sleep with reduced usage. **Conclusions:** Social media use is widely prevalent and commonly perceived to negatively affect mental well-being and sleep, particularly with intensive and late-night use. Self-awareness of these effects may represent a valuable leverage point for prevention, supporting the need for targeted digital wellness strategies and public health interventions.

## 1. Introduction

Social media platforms have become a dominant component of the behavioral environment for young people worldwide, with their use increasing steadily over recent years [1]. Younger individuals frequently spend several hours per day connected to social platforms, fundamentally reshaping patterns of social interaction and information consumption. At the same time, growing concern has emerged regarding the potential implications of this shift for mental health and psychosocial well-being [2].

Several psychological mechanisms may explain the relationship between social media use and mental health outcomes. First, Fear of Missing Out (FoMO) is common among social media users, driven by continuous updates and ephemeral features such as disappearing content that heighten awareness of others’ activities. It is defined as a pervasive apprehension that others experience rewarding events in one’s absence, prompting compulsive social network checking [1,3]. In practice, this pattern is linked to sleep deprivation, emotional tension, and anxiety. Second, social comparison processes are facilitated in online environments. According to social comparison theory, constant exposure to curated images, achievements, and lifestyles of others promotes upward comparisons. Young users may therefore compare themselves unfavorably with idealized portrayals of peers, a process associated with lower self-esteem and increased depressive symptoms [4,5,6,7]. Third, emotional contagion within social networks may amplify mood states. Exposure to emotionally charged content can influence users’ emotional responses, allowing positive or negative affect to spread through online interactions and potentially intensify distress or anxiety [8,9,10]. Social media use has been associated with poorer sleep outcomes in previous studies. Blue light emitted by smartphones and tablets suppresses melatonin secretion and delays sleep onset. In addition, late night social media use reduces sleep duration and increases cognitive and emotional arousal through engaging or stressful content [11,12,13]. Electronic device use at bedtime has consistently been associated with poorer sleep quality and increased daytime sleepiness [14,15].

Although many studies have reported adverse associations between intensive social media use and psychological outcomes [16,17], findings remain heterogeneous. Some studies have reported neutral effects or identified benefits including social connectedness, emotional support, information access, and maintenance of social relationships. Furthermore, the relationship appears to be moderated by factors such as age, motivations for use, patterns of engagement, and sociocultural context [1,17]. Therefore, understanding how individuals themselves perceive the impact of social media may provide complementary insight beyond objective measures of exposure or clinical outcomes.

Conceptually, the present study is informed by Social Comparison Theory and the Health Belief Model. Social Comparison Theory proposes that individuals evaluate themselves through comparisons with others, which may influence emotional well-being when exposure to idealized online content is frequent. The Health Belief Model suggests that individuals’ perceptions of risks and consequences may influence health-related attitudes and behaviors. Together, these frameworks provide a useful basis for understanding how social media users may perceive the influence of online engagement on their emotional well-being and sleep-related experiences [4,6].

Saudi Arabia represents a particularly relevant setting for studying social media use because of its exceptionally high rates of smartphone ownership and social media engagement. Social media platforms play a central role in communication, information exchange, and social participation, notably among young and middle-aged adults. Furthermore, cultural norms emphasizing family connectivity and social interaction may shape both patterns of online engagement and individuals’ perceptions regarding the influence of social media on daily life. These contextual factors may contribute to differences in digital behavior and perceived impacts compared with other populations. In Saudi Arabia, these issues are particularly evident. Internet and smartphone penetration are extremely high, with more than 90% of the population having internet access and most young and middle-aged adults have access in their smartphones [18,19]. Cultural and environmental factors, including extreme heat that limits outdoor activities, encourage indoor and online engagement [18]. Recent surveys report very high levels of social media use among Saudi youth, with 44% of students spending five or more hours daily on these platforms [14]. Problematic internet use in the Gulf region is among the highest worldwide, with pooled prevalence around 33% and estimates in Saudi Arabia reaching 30 to 60% in recent years, coinciding with rising rates of anxiety, depression, and sleep disturbances among the youth [20,21].

Importantly, the construct assessed in this study differs from problematic social media use, FoMO, anxiety, depression, or sleep hygiene. Rather than measuring clinical symptoms or behavioral addiction, the study focuses on users’ subjective perceptions regarding whether and how social media influences their emotions, thoughts, and sleep-related experiences. Such perceptions may provide complementary information regarding digital behavior and self-awareness, even when objective clinical outcomes are not assessed.

Despite the growing literature on digital behavior and mental health, relatively little attention has been given to how users themselves perceive the influence of social media on their psychological well-being and sleep. Yet such subjective perceptions are critical, as they shape behavior, coping strategies, and patterns of platform engagement. Exploring these perceptions may therefore provide complementary insight beyond objective measures of exposure or clinically assessed outcomes. Accordingly, the present study aimed to assess the self-perceived impact of social media use on emotional well-being and sleep-related experiences among healthy young and middle-aged adults residing in Jeddah. Specifically, the study sought to (i) characterize patterns of social media use across major platforms and daily usage behaviors; (ii) evaluate perceived emotional and psychological responses associated with social media engagement; (iii) examine self-reported sleep-related outcomes in relation to social media use; and (iv) identify demographic and behavioral factors associated with greater perceived mental health impact.

## 2. Materials and Methods

### 2.1. Study Design Setting

This study employed a cross-sectional survey among young and middle-aged adults residing in Jeddah, Saudi Arabia. Data were collected using an anonymous online questionnaire distributed through social media platforms and digital networks targeting residents of the city.

### 2.2. Participants

Participants were male and female residents of Jeddah aged 16–50 years. Eligibility was restricted to individuals who reported active use of social media platforms. Participants were excluded if they reported a diagnosed psychiatric disorder, a chronic sleep disorder, or current use of medications known to affect mood or sleep patterns.

### 2.3. Sample Size

The required sample size was estimated using the single population proportion formula for an unknown prevalence of higher perceived mental health impact in an effectively large population. Assuming a conservative prevalence of 50%, a 95% confidence level, and a 5% margin of error, the minimum required sample size was 384 participants. Allowing for incomplete responses and exclusions, recruitment targeted approximately 400 respondents.

### 2.4. Data Collection Tool

Data were collected using a structured self-administered online questionnaire developed for this study. The questionnaire consisted of four sections covering sociodemographic characteristics, social media use patterns, perceived mental health impact, and sleep-related outcomes.

(1) Sociodemographic variables included age, gender, nationality, occupation, educational level, and marital status.
(2) Social media use patterns assessed the use of major platforms (Snapchat, Facebook, WhatsApp, YouTube, TikTok, Instagram, and Twitter/X), daily time spent on social media, time-of-day usage patterns (morning, afternoon, evening, and late night), and use within 30 min before sleep.
(3) Perceived mental health impact was measured using a 6-item Likert-type scale assessing the frequency of emotional or psychological responses to social media exposure, including mood changes, stress or anxiety, social comparison, depressive feelings after scrolling, feelings of isolation, and reduced self-esteem. Items were developed based on themes identified in the previous literature [1,3,4,5,6,7,8,9,10]. Initially, 11 items were generated, which were subsequently reduced to the final six following face and content validity assessment by a panel of experts including two family medicine consultants, one psychiatrist, and one methodologist. Response options ranged from “never/not at all = 0” to “always = 4.” A composite score ranging from 0 to 24 was calculated, with higher scores indicating greater perceived mental health impact. All items were weighted equally.
(4) Sleep-related outcomes were assessed using five items evaluating average sleep duration at night, self-rated sleep quality, insomnia after social media use, frequency of waking up feeling rested, and perceived improvement in sleep after reducing social media use. Items were developed from a self-perception perspective to capture participants’ subjective appraisal of social media’s influence on their sleep, rather than to diagnose or quantify clinical sleep disturbance.

The questionnaire was administered online over a two-month period, with an estimated completion time of approximately 10 min. All questions were set to mandatory, to avoid unanswered questions. Participants were recruited through various social media groups and networks.

### 2.5. Ethical Considerations

Participation in the study was voluntary and anonymous. Informed consent was obtained electronically through the online questionnaire prior to participation. For participants younger than 17 years, parental consent was obtained in accordance with local regulations governing research involving minors. No personally identifiable information was collected. The study was conducted in accordance with the Declaration of Helsinki, and approved by the Institutional Review Board of University of Jeddah (UJ-REC-338 on 8 May 2025).

### 2.6. Statistical Methods

Data were analyzed using SPSS software, version 21 for Windows. Descriptive statistics were used to summarize participant characteristics, social media use patterns, and outcomes: categorical variables were presented as frequencies and percentages, while continuous variables were summarized using means and standard deviations.

Internal consistency of the mental health impact scale was assessed using Cronbach’s alpha. A composite mental health impact score was calculated by summing the six items, with higher scores indicating greater perceived impact. Based on the total score, participants were categorized as having no impact (score 0), low impact (scores 1–11), moderate impact (scores 12–17), or high impact (scores 18–24). For analytical purposes, moderate and high impact (scores 12–24) were grouped and defined as significant mental health impact.

Associations between sociodemographic characteristics, social media use variables, and significant mental health impact were examined using chi-square tests and crude odds ratios (OR) with 95% confidence intervals (CI). All variables that reached statistical significance in unadjusted bivariate analyses were entered simultaneously into the logistic regression model using a forced entry method. Multicollinearity was assessed using the Variance Inflation Factor (VIF); all values were below 5 (range: 1.08–3.77), indicating no evidence of multicollinearity among the predictor variables. Adjusted ORs with 95% CIs were reported, and model performance was assessed using the Nagelkerke R^2^ statistic. Statistical significance was set at *p* < 0.05.

## 3. Results

### 3.1. Participants Flowchart

**Figure 1:**
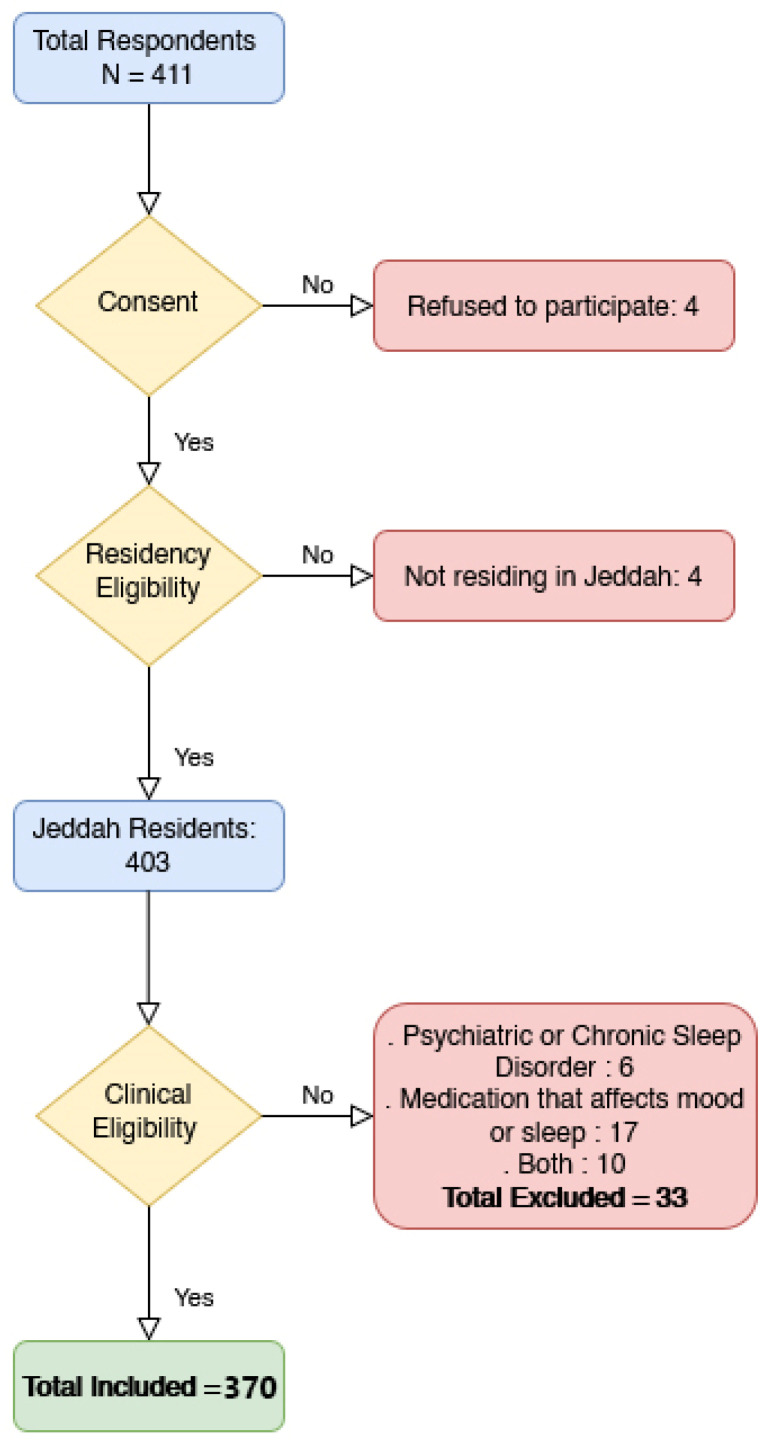
Participants’ Flowchart.

A total of 411 individuals responded to the survey. After exclusion of non-consenting participants, non-residents of Jeddah, and individuals meeting predefined clinical exclusion criteria, 370 participants were retained for the final analysis. Details of the selection process are presented in Figure 1.

### 3.2. Participant Characteristics

Of the 370 participants, nearly half were 18–24 years old (47.6%), and the sample was slightly female predominant (53.5%), with most participants being Saudi (88.9%). Students constituted the majority (51.6%), followed by employed participants (27.8%). Most participants had a bachelor’s degree (45.7%), and 58.9% were single (Table 1).

### 3.3. Social Media Use Patterns

Social media use was widespread (Table 2), with WhatsApp, Snapchat, TikTok, and Instagram emerging as the dominant platforms. Daily engagement was substantial, with most participants reporting between 3 and 6 h of use, and one-fifth exceeding 6 h per day. Use was most frequent in the evening (75.9%), with 44.9% reporting late-night use and 87.6% using social media within 30 min before sleep (Table 2).

### 3.4. Reliability Analysis

The mental health impact scale demonstrated good internal consistency, with a Cronbach’s alpha of 0.866 (6 items). A composite score ranging from 0 to 24 was calculated, with higher scores indicating greater perceived mental health impact. The mean (SD) score was 6.99 (4.89), with observed values ranging from 0 to 24. Most participants reported low impact (scores 1–11; 75.7%), while 5.7% reported no impact, 15.7% moderate impact (12–17), and 3.0% high impact (18–24). Overall, 69 participants (18.6%) were classified as having a significant mental health impact, defined as a score of 12–24.

### 3.5. Perceived Mental Health Impact

Overall, 28.4% reported that social media often or always affected their emotions or mood. In contrast, feelings of stress or anxiety (10.8%), self-comparison (9.4%), depressive feelings after scrolling (8.3%), feelings of isolation (7.6%), and low self-esteem (6.2%) were experienced often or always by smaller proportions of participants (Table 3).

### 3.6. Self-Rated Sleep Outcomes

Most participants reported sleeping 6–7 h per night (53.0%), while 28.1% reported shorter sleep durations (<6 h). Nevertheless, a substantial proportion reported sleep-related difficulties, including insomnia following social media use and infrequent restorative sleep. Notably, nearly half of the participants believed that reducing social media use could improve their sleep, although a comparable proportion remained uncertain (Table 4).

### 3.7. Factors Associated with Perceived Mental Health Impact

Higher perceived impact scores were more frequently observed among younger participants, particularly adolescents and young adults, as well as among students and single individuals. Platform-specific associations were observed for Snapchat, YouTube, and Twitter/X users. Additionally, greater daily social media use and late-night engagement were associated with higher perceived impact scores. No significant associations were identified for gender, nationality, educational level, or most other usage characteristics (Table 5).

### 3.8. Predictors of Perceived Mental Health Impact

In the multivariable logistic regression model (Table 6), participants <18 years had significantly higher odds of reporting higher perceived impact compared with 45–50 years (OR = 11.22, 95% CI: 1.18–106.76; *p* = 0.036). Students also showed higher odds compared with retired or unemployed participants (OR = 2.80, 95% CI: 1.01–7.75; *p* = 0.048); however, the reference category was not significant. Regarding platform use, Snapchat (OR = 2.26, 95% CI: 1.08–4.75; *p* = 0.030) and YouTube (OR = 2.14, 95% CI: 1.11–4.09; *p* = 0.022) were independently associated with increased perceived mental health impact. Late-night social media use was also associated with higher odds (OR = 2.14, 95% CI: 1.12–4.10; *p* = 0.021). The model explained a moderate proportion of variance (Nagelkerke R^2^ = 0.211).

## 4. Discussion

### 4.1. Summary of Findings and Study Approach

The present study explored patterns of social media use among young and middle-aged adults in Jeddah and their perceived impact on mental health and sleep. Participants reporting heavy engagement frequently described negative effects on mood and self-reported sleep-related outcomes. Social media use in Saudi Arabia is largely driven by maintaining contact with friends and family, reflecting strong cultural emphasis on close relationships and highlighting its role in facilitating communication and sustaining social ties. The Saudi sociocultural context may also influence how social media is experienced and perceived. Social media is widely integrated into daily communication and social interaction, potentially increasing both exposure and emotional investment in online activities. Consequently, perceptions regarding the impact of social media on emotional well-being and sleep may be shaped not only by platform characteristics but also by broader social and cultural factors [22].

An important observation was that many participants were able to recognize and articulate the perceived psychological and sleep related effects of their social media use. This self-awareness regarding the consequences of digital habits may represent an important leverage point for prevention. Behavioral health frameworks such as the Health Belief Model suggest that individuals’ perceptions of risks and consequences influence their likelihood of adopting protective behaviors [23]. Thus, when individuals perceive and acknowledge the impact of their media consumption on mood or sleep, they may be more receptive to adopting corrective behaviors, such as moderating usage patterns, reducing nighttime exposure, or seeking guidance when distress becomes persistent. From a public health perspective, promoting this self-awareness through education and digital literacy initiatives may facilitate early identification of harmful patterns and support timely preventive or supportive interventions [24].

### 4.2. Comparison with International and Regional Literature

The findings of the present study align with global evidence. A recent meta-analysis reported small but significant associations between social media use and increased symptoms of depression and anxiety, as well as sleep disturbances. However, substantial heterogeneity was observed across studies, partly explained by factors such as geographical location, anxiety assessment methods, study design, age, and gender, which may act as moderators [17]. Similarly, a prior scoping review concluded that most cross-sectional and longitudinal studies in youth report negative impacts of social media use on sleep and psychological well-being [16]. In our sample, complaints such as increased anxiety, mood fluctuations, and difficulty sleeping were frequently attributed to social media exposure, consistent with international surveys linking excessive online engagement with psychological distress. Woods and Scott [25] reported that adolescents who spent more time using social media, particularly during the evening and night, and those who were more emotionally engaged with these platforms tended to report poorer sleep quality, lower self-esteem, and higher levels of anxiety and depression. Likewise, Abdullah et al. [26] reported a significant association between intensive social media use and poorer sleep quality among university students. Nighttime social media use and engagement with multiple platforms were identified as major contributors to sleep disturbances [26].

Our findings also align with emerging evidence from Saudi Arabia. Alkaabba et al. [14] reported that more than 80% of Saudi university students used screens within 30 min before bedtime, and higher levels of screen exposure were associated with increased anxiety and poorer sleep quality. Similarly, participants in our study frequently identified bedtime device use as an important contributor to perceived sleep disruption. Another Saudi study reported that users of YouTube, Instagram, and Snapchat experienced worse mental health outcomes, with female users in their mid-twenties appearing particularly vulnerable [22]. A cohort study by Alwuqaysi et al. [22] identified TikTok as having particularly negative effects. However, TikTok use was not significantly associated with perceived mental health impact in the present study. This discrepancy may reflect differences in study populations, outcome measures, or patterns of platform use.

Overall, these patterns mirror both global findings and regional evidence, suggesting that intense engagement with image-based and video-based social media may adversely affect perceived well-being.

### 4.3. Demographic Correlates of Perceived Impact

The perceived impact of social media was not uniform across participants. Younger individuals in our sample tended to report more pronounced effects on mood and sleep, consistent with evidence that adolescents and young adults engage more intensively with social media and may be more susceptible to its influence [17]. Age has previously been identified as a moderator in meta-analysis [17]. On the other hand, Abdullah et al. [26] reported no significant association between age and sleep outcomes in their cohort.

Occupation also appeared relevant. Students, who represented the majority of our participants, reported higher screen time and greater levels of perceived anxiety compared with working adults. This observation aligns with previous studies among student populations where intensive social media use has been associated with poorer mental health outcomes [14,26]. Marital status showed a more subtle association. Unmarried participants, predominantly single, perceived greater effects on mental health and sleep, possibly reflecting greater reliance on online social networks for daily interaction. Within the Saudi context, marital and social norms may shape digital communication patterns. Some studies have suggested that single young women report higher levels of FoMO and social media related stress [27].

Although previous studies have reported gender-related differences in social media use and mental health outcomes [28,29], no statistically significant association between gender and perceived mental health impact was observed in the present study. Therefore, our findings do not support the existence of gender differences within this sample. Both Al-Khani et al. [20] and Alwuqaysi et al. [22] documented higher levels of internet addiction and social media related anxiety among young women, a pattern that was not reflected in our data. Conversely, Abdullah et al. [26] did not observe significant gender differences which is consistent with our findings. These variances may reflect differences in usage patterns or in the reporting of distress.

Education may also play a role. Some research suggests that students in demanding academic fields, including health sciences, may engage with social media differently from peers in other disciplines [30].

### 4.4. Platform-Specific Associations

Our analysis indicated that the perceived impact of social media varied across platforms, with Snapchat and YouTube being independently associated with higher perceived impact. These associations should be interpreted with caution, however, as platform use behaviors are likely intercorrelated and confounded by demographic factors, total screen time, and overlapping multi-platform engagement, which the regression model may not fully uncover.

Nonetheless, users of image-based applications frequently report higher levels of stress and anxiety, notably through intensified upward social comparison. Previous literature has suggested that Snapchat’s disappearing-message features may contribute to a sense of urgency among some users, potentially encouraging more frequent engagement. However, these proposed mechanisms were not directly assessed in the present study [1]. Participants in our study similarly described the need to repeatedly check notifications to avoid missing messages, which several linked to sleep interruption and emotional strain [31].

Other visual platforms showed comparable patterns. Abdullah et al. [26] reported that Instagram and TikTok use was associated with poorer sleep quality, and that engagement with multiple platforms further worsened sleep parameters. In contrast, text-oriented platforms such as Twitter/X produced more heterogeneous responses. Although generally perceived as less compulsive than Snapchat, some participants linked Twitter use to increased anxiety, possibly reflecting the rapid and emotionally charged nature of its content. These platform-level differences should nonetheless be regarded as exploratory, given the likelihood of overlapping use across platforms among the same individuals. Experimental evidence indicates that Twitter feeds have been hypothesized to activate brain regions involved in emotional processing more strongly than typical web browsing [32].

YouTube showed a distinct pattern. Many participants used it for passive entertainment, yet heavy users frequently reported prolonged viewing sessions extending late into the night, contributing to poorer sleep the following day [33]. Similar observations were reported by Alwuqaysi et al. [22], who identified YouTube as strongly associated with negative mental health outcomes in a Saudi sample. In contrast, messaging platforms such as WhatsApp were often perceived positively because they mainly facilitated social support. Overall, visual and short-form platforms were more frequently linked to negative outcomes than messaging applications [1].

### 4.5. Timing of Use and Sleep Disruption

Temporal patterns of social media use were critical. Late-night engagement appeared particularly problematic, as participants who remained active on social media late frequently reported poorer sleep and greater next-day fatigue. This observation is consistent with prior studies showing that bedtime screen use disrupts sleep onset and sleep quality [34]. In our sample, 87.6% of participants reported using a device within 30 min before attempting to sleep, a prevalence comparable to that reported in other studies of young populations [14,35]. Participants frequently perceived late-night social media engagement as being associated with difficulty relaxing before sleep. Participants themselves noted that browsing or texting before bedtime kept their minds alert, consistent with mechanisms through which blue light exposure and stimulating content delay melatonin release [34]. Moreover, prolonged screen engagement may displace sleep time, leading individuals to go to bed later and sleep fewer hours. Over time, this may create a self-reinforcing cycle. The relationship between sleep and social media use may be bidirectional. Individuals experiencing sleep difficulties may engage in greater nighttime social media use, while frequent nighttime engagement may also contribute to perceived sleep-related difficulties.

### 4.6. Mechanism of Effect

Although the present study was not designed to investigate underlying mechanisms, participants’ perceptions may be interpreted in the context of theoretical explanations previously proposed in the literature. Exposure to highly arousing content such as news, dramatic videos, and social conflicts late at night can heighten physiological arousal and negative mood, may be perceived as contributing to poorer sleep and mood-related experiences. This is consistent with models of emotional contagion as consuming others’ anxieties or social dramas online can intensify one’s own emotional state [32]. Previous theoretical and behavioral research has proposed that social rewards and social feedback may contribute to repetitive engagement with social media platforms. However, the present study did not assess neurobiological processes and cannot evaluate these proposed mechanisms [3]. Participants often described a compulsive drive to check notifications which a cycle of seeking small hits of social affirmation. Some authors have hypothesized that repeated engagement may reinforce habitual usage patterns; however, such processes cannot be inferred from the current data. In addition, the social comparison process likely exacerbates distress as many users reported feeling worse after scrolling through peers’ idealized posts, exactly as social comparison theory predicts [6,7,36]. When individuals frequently compare themselves to the curated successes of friends or influencers, their self-esteem and mood can decline, which our respondents noted. Collectively, these mechanisms form a feedback loop. Frequent social media use especially at night increases alertness and negative affect, leading to worse sleep and higher stress; in turn, feeling sleep-deprived or anxious encourages even more scrolling seeking distraction or social support, perpetuating the cycle.

### 4.7. Limitations

Several limitations should be considered. First, the cross-sectional design prevents causal inference. Although heavy social media use and bedtime engagement were associated with poorer perceived mental health and sleep, such a relationship may be bidimensional (individuals with anxiety or insomnia may be more likely to engage in online activity). Second, all measures were self-reported, which introduces the possibility of recall and response bias. Participants experiencing distress may overestimate their social media use or attribute negative experiences to it. The use of self-perceived mental health impact, although subjective and potentially requiring clinical validation, nonetheless reflects a more patient-centered and individualized perspective on digital behavior. Such perceptions may capture early experiential signals of distress and encourage self-regulation or corrective behaviors. Third, recruitment via social media platforms using convenience sampling may have introduced selection bias toward active users, limiting the representativeness and generalizability of the findings.

On the other hand, the primary outcome was assessed using a study-specific questionnaire that demonstrated good internal consistency but has not undergone formal psychometric validation. Additionally, the categorization of perceived impact scores into was based on arithmetic thresholds and was not empirically or theoretically validated. Therefore, the findings should be interpreted as exploratory measures of subjective perception—which is the aim of the study—rather than validated indicators of psychological status or sleep quality.

Some regression estimates were associated with wide confidence intervals, particularly within smaller subgroups, indicating limited precision and potential model instability. In addition, certain categories were combined to ensure adequate subgroup sizes for analysis, which may have masked heterogeneity within these groups. These findings should therefore be interpreted cautiously and confirmed in larger studies with greater representation across demographic subgroups.

Finally, because of the cross-sectional design, it is not possible to establish temporal directionality or distinguish cause from effect. The observed associations may reflect a bidirectional relationship in which psychological distress influences social media use, social media use influences perceptions of distress, or both. Future studies should complement subjective assessments with objective indicators, such as platform usage logs or actigraphy-based sleep monitoring, and consider additional factors including offline social support and personality traits.

### 4.8. Future Directions and Public Health Implications

The findings of the present study highlight several implications for research and prevention. Notably, self-awareness of digital habits may represent an important leverage point for early prevention and behavioral adjustment. Building on these observations, several directions for research and public health action can be considered:

- Longitudinal cohort studies in Saudi Arabia and other settings are needed to clarify causal pathways. Following individuals over time would help determine whether intensive evening social media use precedes deterioration in sleep and mental health, or whether existing psychological distress promotes greater online engagement.
- Interventional studies should evaluate whether modifying digital behavior improves outcomes. Randomized trials examining reduced nighttime social media use or digital curfews could determine whether sleep quality and emotional well-being improve when evening screen exposure is limited.
- Future research should diversify measurement approaches by complementing self-reported perceptions with objective indicators such as platform usage logs, wearable sleep monitoring devices, and validated psychological assessment tools.
- From a public health perspective, digital wellness education is essential. Increasing awareness of the potential sleep disrupting effects of late-night screen exposure may encourage concerned individuals to adopt healthier sleep hygiene practices, including limiting device use before bedtime.
- Promoting healthier social media habits may also be beneficial. Educational initiatives may encourage moderation of daily use, thoughtful curation of online content to reduce negative comparisons, and boundaries for notifications and nighttime engagement.
- Universities and healthcare institutions in Saudi Arabia could implement workshops or awareness campaigns on balanced technology use, using users’ self-awareness as an entry point to promote preventive behaviors and early corrective measures.

## 5. Conclusions

Social media use was highly prevalent among participants, with substantial engagement during evening and late-night hours. A considerable proportion of respondents perceived that social media use, particularly late-night engagement and use of certain platforms, was associated with negative emotional experiences and sleep-related difficulties. Because these findings are based on self-reported perceptions measured using a study-specific questionnaire, they should not be interpreted as evidence of clinically confirmed mental health impairment or objectively measured sleep disturbances. The findings highlight the importance of understanding how individuals perceive the influence of digital behaviors on their well-being and support the need for longitudinal studies employing validated psychological and sleep assessment instruments to further clarify these relationships.

## Figures and Tables

**Table 1:** Participant characteristics (N = 370).

Parameter	Level	N	%
Age (year)	<18	26	7.0
	18–24	176	47.6
	25–34	38	10.3
	35–45	61	16.5
	45–50	69	18.6
Gender	Male	172	46.5
	Female	198	53.5
Nationality	Saudi	329	88.9
	Non-Saudi	41	11.1
Occupation	Student	191	51.6
	Employed	103	27.8
	Unemployed	66	17.8
	Retired	10	2.7
Educational level	High school	132	35.7
	Diploma	29	7.8
	Bachelor’s degree	169	45.7
	Master’s degree	24	6.5
	PhD	16	4.3
Marital status	Single	218	58.9
	Married	138	37.3
	Divorced	13	3.5
	Widowed	1	0.3

**Table 2:** Social media use patterns (N = 370).

Parameter	Level	N	%
Snapchat use	No	120	32.4
	Yes	250	67.6
Facebook use	No	340	91.9
	Yes	30	8.1
WhatsApp use	No	100	27
	Yes	270	73
YouTube use	No	177	47.8
	Yes	193	52.2
TikTok use	No	132	35.7
	Yes	238	64.3
Instagram use	No	150	40.5
	Yes	220	59.5
Twitter/X use	No	247	66.8
	Yes	123	33.2
Daily time spent on social media	<1 h	6	1.6
	1–2 h	44	11.9
	3–4 h	138	37.3
	5–6 h	108	29.2
	>6 h	74	20.0
Frequent morning use	No	241	65.1
	Yes	129	34.9
Frequent afternoon use	No	217	58.6
	Yes	153	41.4
Frequent evening use	No	89	24.1
	Yes	281	75.9
Frequent late-night use	No	204	55.1
	Yes	166	44.9
Use within 30 min before sleep	No	46	12.4
	Yes	324	87.6

**Table 3:** Mental health assessment.

Dimension	Always	Often	Sometimes	Rarely	Never/Not at All
Social media affecting emotions or mood	38 (10.3)	67 (18.1)	177 (47.8)	51 (13.8)	37 (10.0)
Stressed or anxious after using social media	11 (3.0)	29 (7.8)	125 (33.8)	87 (23.5)	118 (31.9)
Self-comparison on social media	6 (1.6)	29 (7.8)	78 (21.1)	85 (23.0)	172 (46.5)
Depression after scrolling on social media	9 (2.4)	22 (5.9)	94 (25.4)	109 (29.5)	136 (36.8)
Isolation after seeing others’ posts	8 (2.2)	20 (5.4)	70 (18.9)	79 (21.4)	193 (52.2)
Low self-esteem after using social media	9 (2.4)	14 (3.8)	70 (18.9)	81 (21.9)	196 (53.0)

**Table 4:** Self-reported sleep-related outcomes assessment.

Parameter	Level	N	%
Hours of sleep	<4 h	19	5.1
	4–5 h	85	23.0
	6–7 h	196	53.0
	≥8 h	70	18.9
Self-reported sleep-related outcomes rating	Very poor	14	3.8
	Poor	79	21.4
	Fair	122	33.0
	Good	116	31.4
	Excellent	39	10.5
Insomnia after using social media	No	254	68.6
	Yes	116	31.4
Waking up feeling rested	Never	15	4.1
	Rarely	77	20.8
	Sometimes	158	42.7
	Often	96	25.9
	Always	24	6.5
Reducing social media use improves sleep	No	50	13.5
	Yes	164	44.3
	Maybe	156	42.2

**Table 5:** Factors associated with higher perceived mental health impact.

Parameter	Level	Higher Perceived Impact, N (%)	*p*-Value	Unadjusted OR (95% CI)
Age (year)	45–50	5 (7.2)	0.004 *	Ref
	35–45	7 (11.5)		1.67 (0.49–5.71)
	18–24	37 (21.0)		3.40 (1.28–9.01)
	25–34	11 (28.9)		5.23 (1.63–16.76)
	<18	9 (34.6)		6.80 (2.01–23.03)
Gender	Female	35 (17.7)	0.607	Ref
	Male	34 (19.8)		1.15 (0.68–1.95)
Nationality	Saudi	60 (18.2)	0.565	Ref
	Non-Saudi	9 (22.0)		1.26 (0.57–2.78)
Occupation	Retired/unemployed	7 (9.2)	0.042 *	Ref
	Employed	19 (18.4)		2.22 (0.90–5.48)
	Student	43 (22.5)		2.87 (1.24–6.64)
Educational level	PhD	2 (12.5)	0.142	Ref
	Bachelor	23 (13.6)		1.10 (0.24–5.04)
	Diploma	6 (20.7)		1.82 (0.34–9.76)
	Master	5 (20.8)		1.83 (0.31–10.78)
	High school	33 (25.0)		2.33 (0.51–10.68)
Marital status	Married	16 (11.6)	0.027 *	Ref
	Divorced/widowed	3 (21.4)		2.09 (0.54–8.06)
	Single	50 (22.9)		2.27 (1.20–4.31)
Snapchat use	No	12 (10.0)	0.003 *	Ref
	Yes	57 (22.8)		2.66 (1.36–5.19)
Facebook use	Yes	3 (10.0)	0.205	Ref
	No	66 (19.4)		2.17 (0.64–7.33)
WhatsApp use	No	18 (18.0)	0.845	Ref
	Yes	51 (18.9)		1.06 (0.58–1.93)
YouTube use	No	20 (11.3)	0.001 *	Ref
	Yes	49 (25.4)		2.67 (1.51–4.73)
TikTok use	No	21 (15.9)	0.314	Ref
	Yes	48 (20.2)		1.34 (0.76–2.36)
Instagram use	No	27 (18.0)	0.791	Ref
	Yes	42 (19.1)		1.08 (0.62–1.88)
Twitter/X use	No	37 (15.0)	0.010 *	Ref
	Yes	32 (26.0)		2.00 (1.18–3.41)
Time spent on social media	≤2 h	4 (8.0)	<0.001 *	Ref
	3–4 h	19 (13.8)		1.84 (0.59–5.71)
	5–6 h	20 (18.5)		2.60 (0.84–8.06)
	>6 h	26 (35.1)		6.21 (2.01–19.16)
Morning use	No	41 (17.0)	0.269	Ref
	Yes	28 (21.7)		1.36 (0.80–2.32)
Afternoon use	No	37 (17.1)	0.347	Ref
	Yes	32 (20.9)		1.28 (0.76–2.18)
Evening use	No	13 (14.6)	0.261	Ref
	Yes	56 (19.9)		1.45 (0.75–2.80)
Late-night use	No	23 (11.3)	<0.001 *	Ref
	Yes	46 (27.7)		2.99 (1.70–5.25)
Social media ≤30 min before sleep	No	6 (13.0)	0.297	Ref
	Yes	63 (19.4)		1.61 (0.66–3.94)

The *p*-value corresponds to overall group comparison using chi square test. * Statistically significant difference (*p* < 0.05).

**Table 6:** Predictors of higher perceived mental health impact.

Predictor	Category	OR	95% CI	*p*-Value
Age (years)	45–50	Ref	-	0.184
	<18	11.22	1.18–106.76	0.036 *
	18–24	5.74	0.75–43.87	0.092
	25–34	3.83	0.93–15.82	0.063
	35–45	1.32	0.38–4.63	0.663
Occupation	Retired/unemployed	Ref	-	0.062
	Employed	0.73	0.14–3.74	0.707
	Student	2.80	1.01–7.75	0.048 *
Marital	Married	Ref	-	0.774
	Single	0.74	0.19–2.83	0.661
	Divorced/widowed	0.57	0.11–2.88	0.497
Snapchat	No	Ref	-	-
	Yes	2.26	1.08–4.75	0.030 *
YouTube	No	Ref	-	-
	Yes	2.14	1.11–4.09	0.022 *
Twitter/X	No	Ref	-	-
	Yes	1.04	0.54–1.99	0.914
Time spent	≤2 h	Ref	-	0.114
	3–4 h	1.01	0.30–3.38	0.983
	5–6 h	1.25	0.37–4.25	0.726
	>6 h	2.42	0.69–8.41	0.166
Late-night use	No	Ref	-	-
	Yes	2.14	1.12–4.10	0.021 *

Nagelkerke R Square = 0.211. * Statistically significant difference (*p* < 0.05).

## Data Availability

The data supporting the findings of this study are available from the corresponding author upon reasonable request. Due to ethical and privacy considerations, the data are not publicly available.
